# Trends in NLRP3 inflammasome research in ischemic stroke from 2011 to 2022: A bibliometric analysis

**DOI:** 10.1111/cns.14232

**Published:** 2023-04-23

**Authors:** Hua Zhu, Yonggang Zhang, Shi Feng, Yina Li, Yingze Ye, Zhihong Jian, Xiaoxing Xiong, Lijuan Gu

**Affiliations:** ^1^ Department of Neurosurgery Renmin Hospital of Wuhan University Wuhan China; ^2^ Central Laboratory Renmin Hospital of Wuhan University Wuhan China

**Keywords:** bibliometric analysis, ischemic stroke, NLRP3, visualization analysis, web of science

## Abstract

**Background:**

Ischemic stroke is a leading cause of permanent disability and death globally. The nucleotide‐biding oligomaerization domain (NOD)‐like receptor family pyrin domain‐containing 3 (NLRP3) inflammasome is a multi‐protein complex that plays a role in ischemic stroke. Recently, research on the role of NLRP3 in ischemic stroke has developed rapidly worldwide. However, there is no bibliometric analysis of NLRP3 in ischemic stroke to date.

**Aim:**

Through bibliometric analysis, the aim of this study was to assess the current state of research on NLRP3 in the field of ischemic stroke research worldwide over the past 12 years and to identify important results, major research areas, and emerging trends.

**Methods:**

Publications related to NLRP3 in ischemic stroke from January 1, 2011 to December 31, 2022 were obtained from the Web of Science Core Collection (WoSCC). We used HistCite, VOSviewer, CiteSpace, and Bibliometrix for bibliometric analysis and visualization. The Total Global Citation Score (TGCS) was employed to assess the impact of publications.

**Results:**

We found that research of NLRP3 in ischemic stroke developed rapidly starting in 2011. 601 relevant studies have been published in 245 journals over the past 12 years. *Journal of Neuroinflammation* and *International Immunopharmacology* were the most productive journals and *Journal of Neuroinflammation* was the most cited journal. Additionally, *Stroke* and *Journal of Cerebral Blood Flow & Metabolism* were the most co‐cited journal. The most productive country was China (records = 430) and the most productive university was the Zhejiang University (records = 24). *Arumugam TV* (TGCS = 949) was the most cited author in this field. NLRP3 inflammasome activation, nf–κb, oxidative stress, and inflammation were the knowledge bases for the research in this field.

**Conclusion:**

This study is a scientometric study utilizing quantitative and qualitative methods to comprehensively review the publications on NLRP3 in ischemic stroke. This information provides a reference for scholars to further study NLRP3 in ischemic stroke.

## Graphical abstract

In this work, we HistCite Pro 2.1, VOSviewer, CiteSpace software, and biblioshiny to analyzed 601 publications on NLRP3 in ischemic stroke using a bibliometric approach and systematically evaluated the status of recent NLRP3 research in ischemic stroke injury.

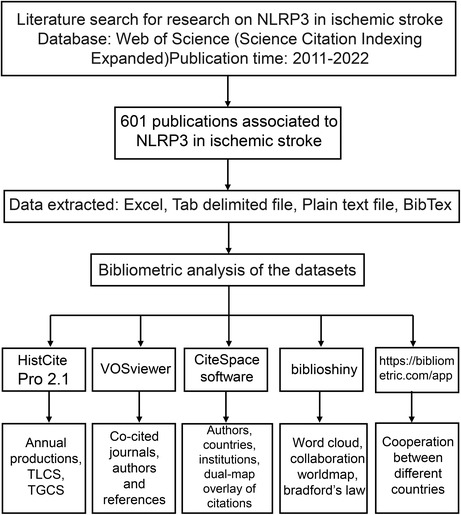

## 1 INTRODUCTION

Stroke remains the leading cause of death and long‐term disability worldwide, with ischemic stroke accounting for approximately 85% of all cases.^1^, ^2^ Ischemic stroke is a disease induced by cerebral blood flow cessation. The treatment of ischemic stroke is based on the restoration of blood flow in the ischemic area.^3^ Thrombolytic treatment is the treatment of choice for acute ischemic stroke. Many patients with ischemic stroke, however, are not eligible for this treatment because of the narrow time window for treatment and the risk of cerebral hemorrhage.^4^ In addition, the process of reconstitution of blood flow can lead to further damage of ischemic tissue through infiltration of neutrophils, disturbance of cellular ion homeostasis, accumulation of ROS, and a subsequent inflammatory response leading to cell death.^5^ Although significant advancement has been made in the last few decades in neuroprotective therapies and the salvage of dead neurons, and rehabilitation methods have been developed to address poststroke deficits; no significant progress has been made in the treatment and clinical recovery of stroke.^6^ Occlusion of a major vessel such as the internal carotid artery (ICA) or the middle cerebral artery (MCA) leads to an immediate cessation of oxygen and glucose supply and rapid progression to infarction in the MCA region unless recanalization is achieved in time. Hypoxic cells of the ischemic parenchyma, mainly neurons, respond by upregulating and secreting danger signals, called damage‐associated molecular patterns (DAMPs); which themselves trigger secondary signaling cascades. Postischemic aseptic inflammation has both protective and deleterious effects on disease progression. Overall, the molecular mechanisms of postischemic neuronal inflammatory injury are complex and yet to be fully investigated.^7^

In recent years, researchers have recognized a new inflammasome signaling pathway, NLRP3 inflammasome containing receptor 3, as a potentially important mediator for detecting cellular injury and mediating post‐stroke inflammation.^8^, ^9^, ^10^ NOD‐like receptors (NLRs) are pathogen‐recognition receptors expressed mainly in the cytoplasm that can detect signals from intracellular invaders.^11^ There are different categories of NLRs that form the inflammasome; including NLRP1, NLRP3, NLRP6, NLRP7, NLRP12, NLRC4, NLRC5, and AIM2.^12^ Among these inflammasomes, NLRP3 is the most characteristic, encoded by the cold‐induced autoinflammatory syndrome‐1 (CIAS‐1) gene, and highly expressed in neuronal cells and immune cells.^13^, ^14^ The NLRP3 inflammasome is also the most widely investigated and is thought to be closely associated with aseptic inflammation that is mainly located in the cytoplasm.^15^, ^16^ The NLRP3 protein, the adapter protein ASC, and the inflammatory caspase‐1 are three sections in the NLRP3 inflammasome.^17^ Multiple mechanisms regulate NLRP3 inflammatory vesicles after ischemic stroke.^18^ ROS production after ischemic stroke can trigger neuronal cell damage, brain edema, and neurological deficits by stimulating the brain inflammatory response and NLRP3 inflammasome.^5^, ^19^ Suppression of the NLRP3 inflammasome pathway is thought to exert neuroprotective effects in ischemic stroke models. Research in the role of NLRP3 in ischemic stroke is active and promising, therefore an analysis of the hot spots and trends within the field is warranted.

Bibliometrics is a convenient and fast new method for qualitative research and quantitative analysis of publications.^20^ It focuses on the metrological features of literature, and identifies different characteristics such as countries, institutions, journals, authors, and keywords for various publications in a given field over a period of time^21^; enabling researchers to summarize the current status and developmental trends of a research field or a specific disease, and provide direction and ideas for future research.^22^ Scientometric analysis generally consists of three steps: (1) obtaining literature from accessible databases; (2) analyzing it through software tools, and (3) writing the manuscript. With this method, researchers can quickly dig deeper into the evolution of topics, major research areas, and new research directions in a particular research area.^23^ Bibliometrics as a complementary research method that has been widely used in many disciplines.^24^ However, bibliometric studies of NLRP3 in ischemic stroke are still lacking.

In this study, we analyzed publications on NLRP3 in ischemic stroke using a bibliometric approach and systematically evaluated the status of recent NLRP3 research in ischemic stroke injury, current research priorities, and new research trends for systematic evaluation; highlighting landmark results and pointing out future research directions.

## 2 METHODS

There are no animal studies, no human studies, no potentially identifiable human images, or data presented in this manuscript; therefore, ethical approval is not required in this work.

### 2.1 Data source and search strategy

The Web of Science Core Collection (WoSCC) was employed to obtain studies related to NLRP3 in ischemic stroke from January 1, 2011 to December 31, 2022. To obtain studies on NLRP3‐related ischemic stroke in the past 12 years, we used the following search strategy: TOPIC: (“NLRP3”) AND (TOPIC (“ischemic stroke” OR “cerebral ischemia” OR “ischaemic stroke” OR “brain ischemia” OR “cerebral ischaemia” OR “cerebral infarction” OR “brain ischaemia” OR “brain infarction”)).^25^ The language in our search is restricted to English. The type of literature is limited to articles and reviews. All the eligible data were downloaded from the WoSCC and further analyzed by scientometric tools.

### 2.2 Statistical analysis

HistCite Pro 2.1^26^ was used to calculate the records, total local citation score (TLCS), and total global citation score (TGCS) of each publication, journal, country, productive institution, and author. We used VOSviewer (1.6.13) to identify highly productive countries/regions, institutions, journals and authors, as well as major co‐cited journals, authors and references; and to construct relevant visualization networks.^27^ The link between nodes indicated the co‐occurrence relationship, and the size of the link indicated the co‐occurrence frequency of two nodes. We used CiteSpace V software to explore the trends and dynamics of scientific research in research related to NLRP3 in ischemic stroke, as well as visualize and analyze knowledge domains and emerging trends^20^; including network of authors, countries, institutions, co‐cited authors and references, and dual‐map overlay of citations.^23^, ^28^ Cluster analysis timeline views of clusters were performed on the keywords, and the clusters were named according to the extracted keywords. In addition, we identified keywords with strong citation bursts by CiteSpace V. R package “bibliometrix” and Rstudio software (version 2022.12.0 + 353) were employed to explore the word cloud of keywords, collaboration worldmaps, core journals based on Bradford's law (Bradford's law can be used to describe the dispersion of citations in a topic or field and to identify the most cited journals in a field or discipline^29^), three‐field plots, annual scientific production worldmaps, and co‐cited journals with most TGCS. Another web tool (https://bibliometric.com/app) was used for analyzing the cooperation between different countries.

## 3 RESULTS

### 3.1 Annual publications analysis

**FIGURE 1:**
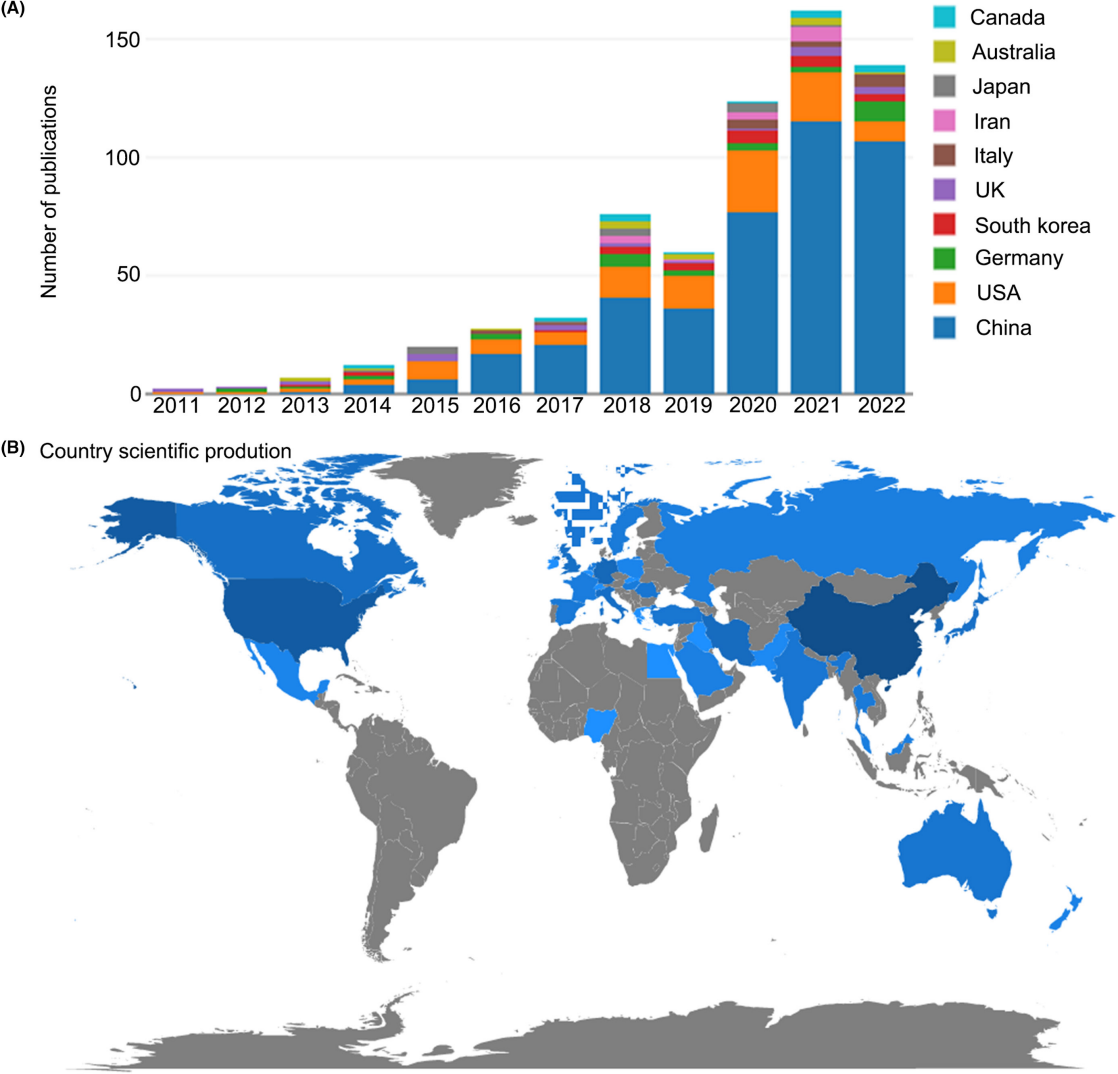
Annual output of research related to NLRP3 in ischemic stroke (A) and country/region scientific production world map of NLRP3 research in ischemic stroke (B).

A total of 601 publications associated with NLRP3 in ischemic stroke were retrieved from WoSCC, 453 articles, and 141 reviews (Table S1). Starting from 2011, the number of papers published in this field increased from 2 to 143, with rapid growth in the number of papers published annually (Figures 1A, S1, Table S2). The average annual production was about 50 publications/year, with an average of less than 10 publications/year from 2011 to 2014, and a continuous increase from 19 to 55 between 2015 and 2019. Annual production exceeded 100 publications for the first time in 2020 (*n* = 103, 17.88%) and peaked in 2021 (*n* = 143, 24.83%). By December 31, 2022, the number of publications in the field was 137 in 2022.

### 3.2 Leading countries

Among the 601 publications we obtained, the corresponding authors were distributed in 37 countries or regions, as illustrated in Figure 1B. On the world map, a country or region in blue means that there were corresponding authors from there, and the intensity of the blue color was proportional to the number of publications in that country or region; the darker the blue color, the more publications were published. China, the United States, and Germany had the largest number of publications, accounting for 71.55%, 17.80%, and 4.33%, respectively. In addition to these three countries, there were some other countries with more than 10 publications; including South Korea, UK, Australia, Iran, Italy, Japan, and Canada. The top 5 countries in terms of number of articles published were China, USA, Germany, South Korea, and UK (Table 1). Additionally, the top 10 most cited countries were China, USA, Germany, Australia, Singapore, South Korea, UK, Canada, Japan, and Italy (Table 1).

**TABLE 1:** Top 10 productive countries and most cited countries.

Rank	Most productive countries				Most cited countries			
	Country	Records	TLCS	TGCS	Country	Records	TLCS	TGCS
1	Peoples R China	430	1096	9870	Peoples R China	430	1096	9870
2	USA	107	242	3891	USA	107	242	3891
3	Germany	26	105	1486	Germany	26	105	1486
4	South Korea	22	132	954	Australia	13	218	1074
5	UK	20	112	813	Singapore	8	190	1049
6	Italy	14	0	583	South Korea	22	132	954
7	Australia	13	218	1074	UK	20	112	813
8	Canada	13	47	760	Canada	13	47	760
9	Iran	13	78	472	Japan	13	17	629
10	Japan	13	17	629	Italy	14	0	583

**FIGURE 2:**
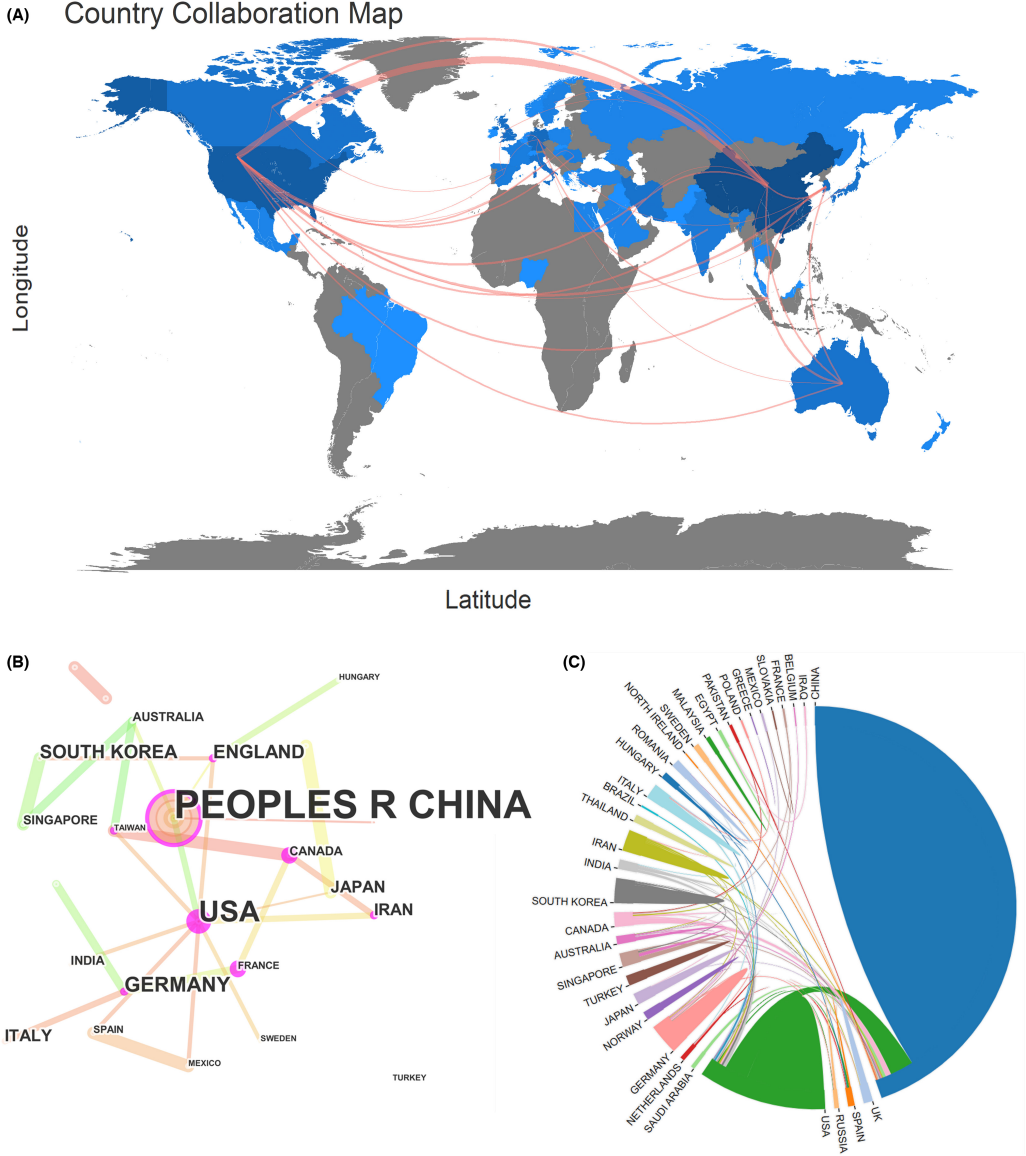
Cooperation between countries. (A) Country or region collaboration world map of NLRP3 research in ischemic stroke. (B, C) The network map of countries for NLRP3 research in ischemic stroke.

Figure 2A shows a map of national or regional collaboration on NLRP3 in the field of ischemic stroke. If there was some kind of collaboration between two countries or regions, they would be connected by a red line, the thickness of which is proportional to the number of collaborations. As shown in Figure 2A, mainland China and the United States were the central countries of the publications. There were collaborations between various countries. In particular, the collaboration between the United States and mainland China has 43 co‐published papers (Table S3). Additionally, through using the CiteSpace software and an online bibliometric app, the similar results of collaboration between different countries were presented in Figure 2B,C.

### 3.3 Active institutions

**FIGURE 3:**
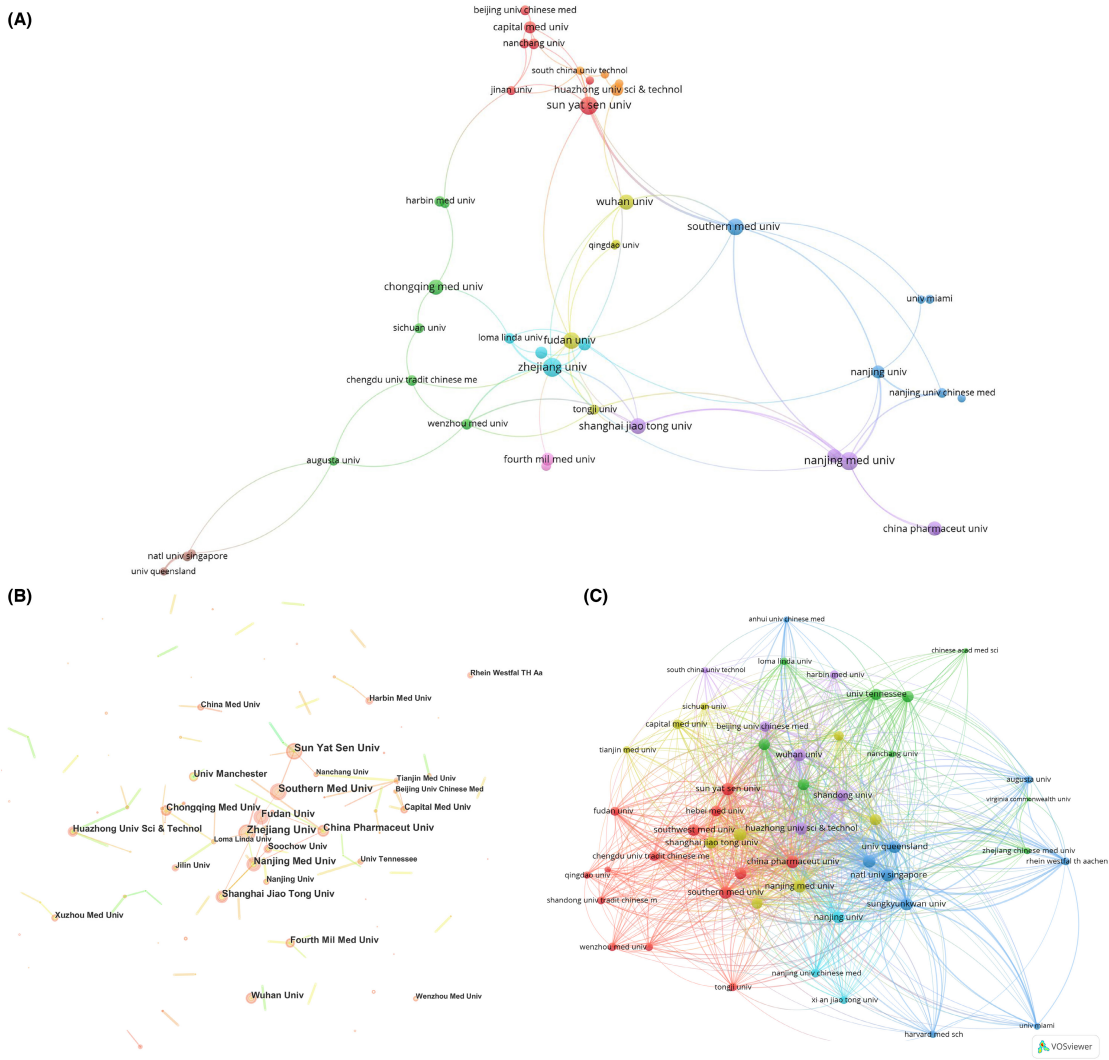
The network map of institutions. (A, B) Coauthorship between institutions in NLRP3 research in ischemic stroke. (C) Citation analysis of organizations in NLRP3 research in ischemic stroke.

A total of 3025 authors from 706 institutions published articles on NLRP3 in relation to cerebral ischemic stroke. The top 10 institutions with the highest research output within the field are shown in Table 2. The Zhejiang University (*n* = 24) was the leading institutions in terms of output, followed by the Nanjing Medical University (*n* = 22), Sun Yat Sen University (*n* = 22), Southern Medical University (*n* = 20), Fudan University (*n* = 19), and Shanghai Jiao Tong University (*n* = 18). Natl Univ Singapore had the highest TGCS (1031 citations), followed by Sun Yat‐sen University (836 citations) and Univ Queensland (827 citations). Collaboration among institutions was relatively close. Zhejiang University, Nanjing Medical University, and Southern Medical University were the most collaborative with other institutions (Figure 3A–C).

**TABLE 2:** Top 10 most productive and cited institutions.

Rank	Most productive institutions				Most cited institutions			
	Institution	Records	TLCS	TGCS	Institution	Records	TLCS	TGCS
1	Zhejiang Univ	24	64	789	Natl Univ Singapore	7	190	1031
2	Nanjing Med Univ	22	144	676	Sun Yat Sen Univ	22	37	836
3	Sun Yat Sen Univ	22	37	836	Univ Queensland	5	159	827
4	Southern Med Univ	20	11	500	Zhejiang Univ	24	64	789
5	Fudan Univ	19	11	239	Sungkyunkwan Univ	5	108	727
6	Shanghai Jiao Tong Univ	18	85	470	Monash Univ	3	130	725
7	Chongqing Med Univ	16	131	674	Nanjing Med Univ	22	144	676
8	Wuhan Univ	16	14	516	Chongqing Med Univ	16	131	674
9	China Pharmaceut Univ	14	97	648	China Pharmaceut Univ	14	97	648
10	Xuzhou Med Univ	13	75	353	Univ Manchester	12	96	578

### 3.4 Core journals and co‐cited journals

**FIGURE 4:**
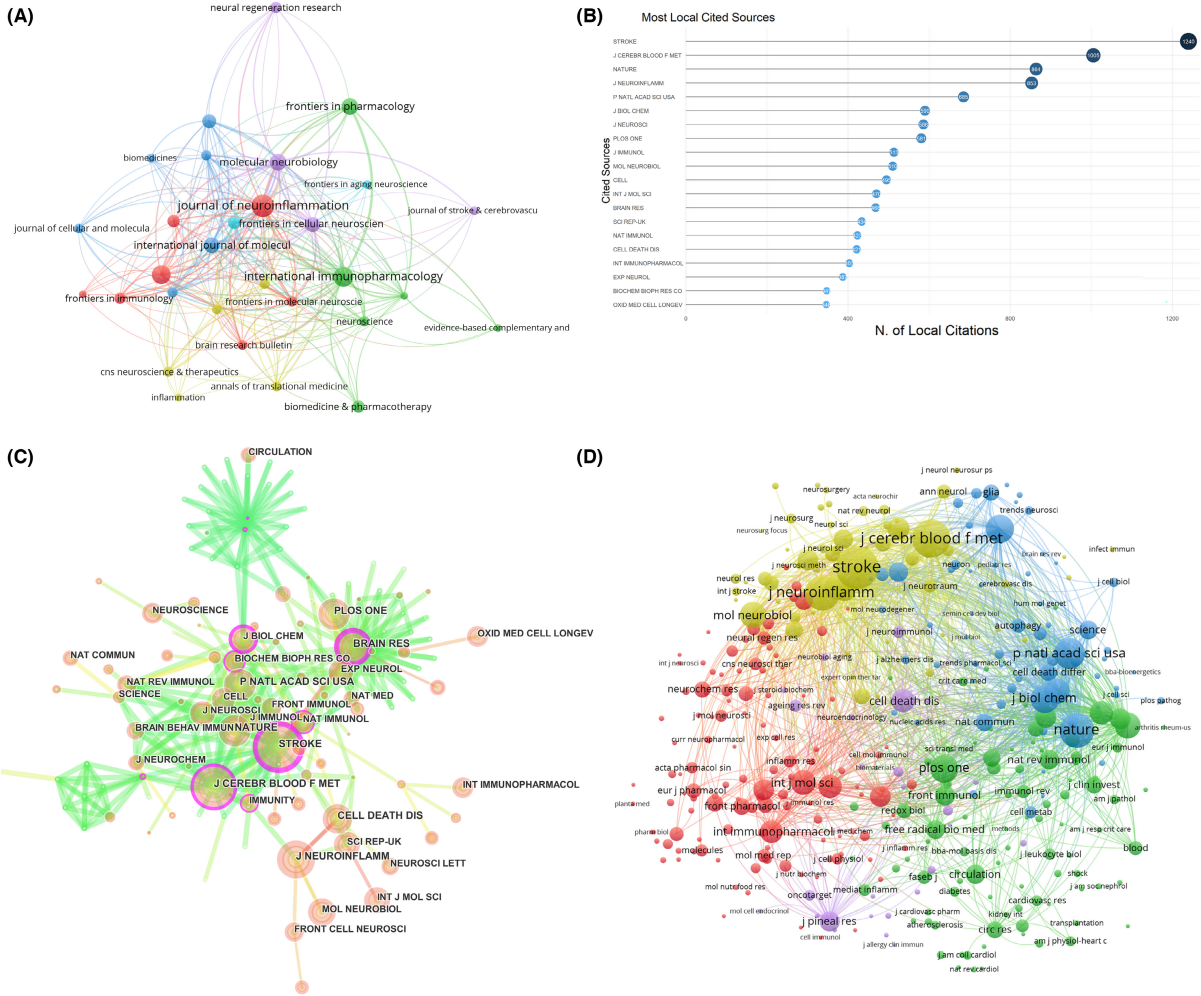
The network of journals and cocited journals. (A) Citation analysis of journals for NLRP3 research in ischemic stroke. (B) Top 20 most cited cocited journals. (C, D) Network map of cocited journals for NLRP3 research in ischemic stroke performed by CiteSpace and VOSviewer, respectively.

**FIGURE 5:**
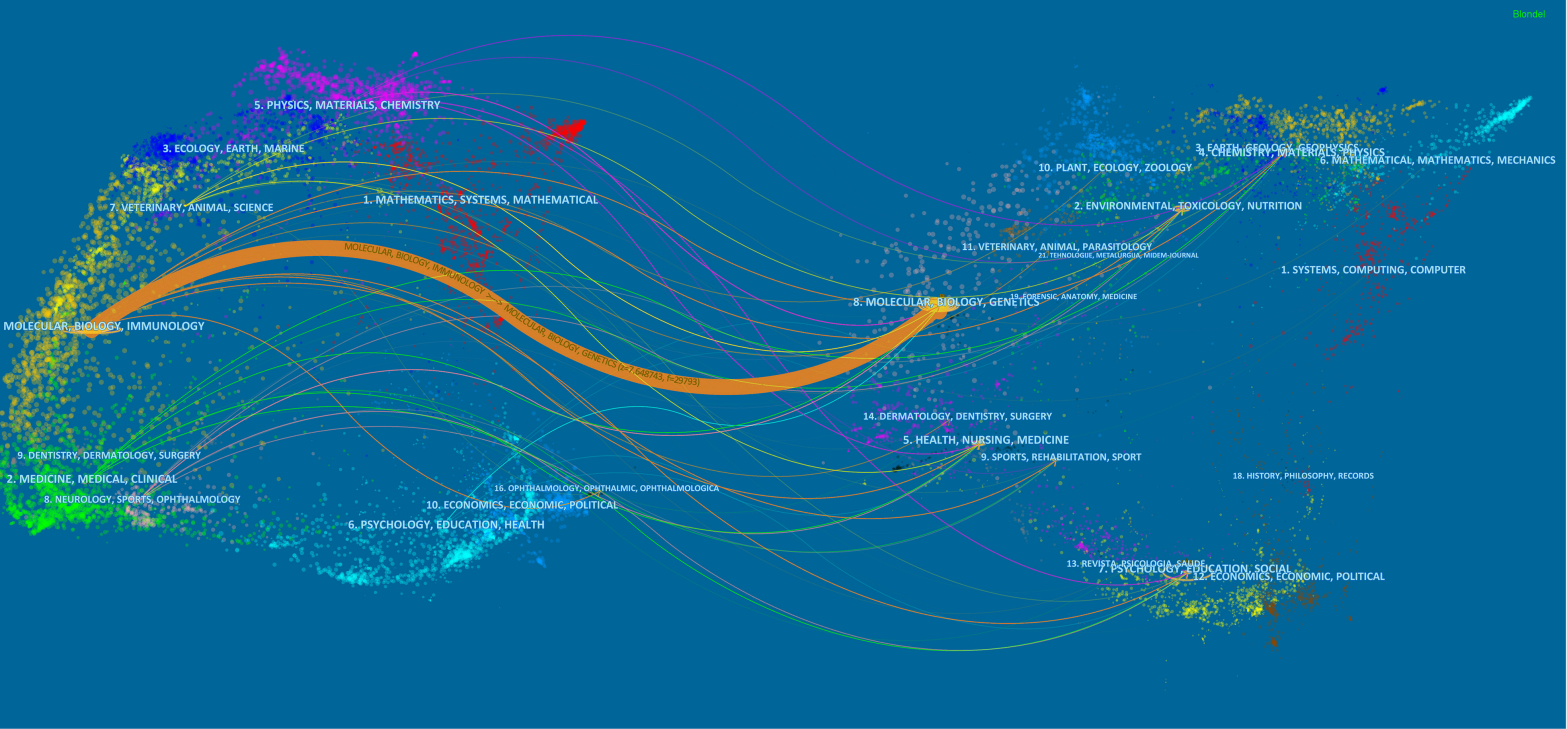
The dual‐map overlay of journals associated with NLRP3 research in ischemic stroke.

The literature related to NLRP3 in ischemic stroke was published in 245 journals. The 10 journals with the highest productivity are shown in Table 3. 25% of the literature was published in these journals. The journal with the highest number of publications was *Journal of neuroinflammation* (23 publications), followed by *International immunopharmacology* (19 publications published) and *Oxidative medicine and cellular longevity* (18 publications). Figure 4A shows that *Journal of neuroinflammation*, *International immunopharmacology*, *Oxidative medicine and cellular longevity and Frontiers in pharmacology* published the most papers. *Journal of neuroinflammation* had active citation relationships with *Neuroscience*, *Frontiers in neuroscience*, *Frontiers in pharmacology*, and *Journal of stroke & cerebrovascular diseases*. *International immunopharmacology* had citation relationships with journals such as *biomedicine & pharmacotherapy* and *Neuroscience*. The top 5 most cited journals were *Journal of neuroinflammation* (TGCS = 985), *International immunopharmacology* (TGCS = 598), *Oxidative medicine and cellular longevity* (TGCS = 568), *Molecular neurobiology* (TGCS = 563), and *Cell death & disease* (TGCS = 556) (Table 3). According to the Bradford's law, the top 16 core journal were presented in Figure S2. *Journal of neuroinflammation* and *International immunopharmacology* were the top 2 core journals in this field. The top 20 most co‐cited journals were showed in Figure 4B. Among the co‐cited academic journals; *Stroke* had the most co‐citations, followed by *Journal of cerebral blood flow and metabolism*, *Nature*, *Journal of Neuroinflammation*, and *Proc Natl Acad Sci USA* (Figure 4B). The network visualization of co‐cited journals was presented in Figure 4C,D. The dual‐map showed one major citation pathway. The published articles were mainly concentrated in journals in the fields of molecules, biology, and immunology; while the cited publications were mainly published in the fields of molecules, biology, and genetics (Figure 5).

**TABLE 3:** Top 10 productive journals and top 10 most cited journals.

Rank	Most productive journals	Records	TGCS	Most cited journals	Records	TGCS
1	Journal of neuroinflammation	23	985	Journal of neuroinflammation	23	985
2	International immunopharmacology	19	598	International immunopharmacology	19	598
3	Oxidative medicine and cellular longevity	18	568	Oxidative medicine and cellular longevity	18	568
4	Frontiers in pharmacology	15	179	Molecular neurobiology	15	563
5	Molecular neurobiology	15	563	Cell death & disease	4	556
6	International journal of molecular sciences	14	418	Brain behavior and immunity	7	464
7	Frontiers in cellular neuroscience	13	388	Nature reviews neuroscience	1	444
8	Biochemical and biophysical research communications	12	296	International journal of molecular sciences	14	418
9	Biomedicine and pharmacotherapy	10	189	Experimental neurology	10	414
10	Experimental neurology	10	414	Frontiers in cellular neuroscience	13	388

### 3.5 Active authors and co‐cited authors

**FIGURE 6:**
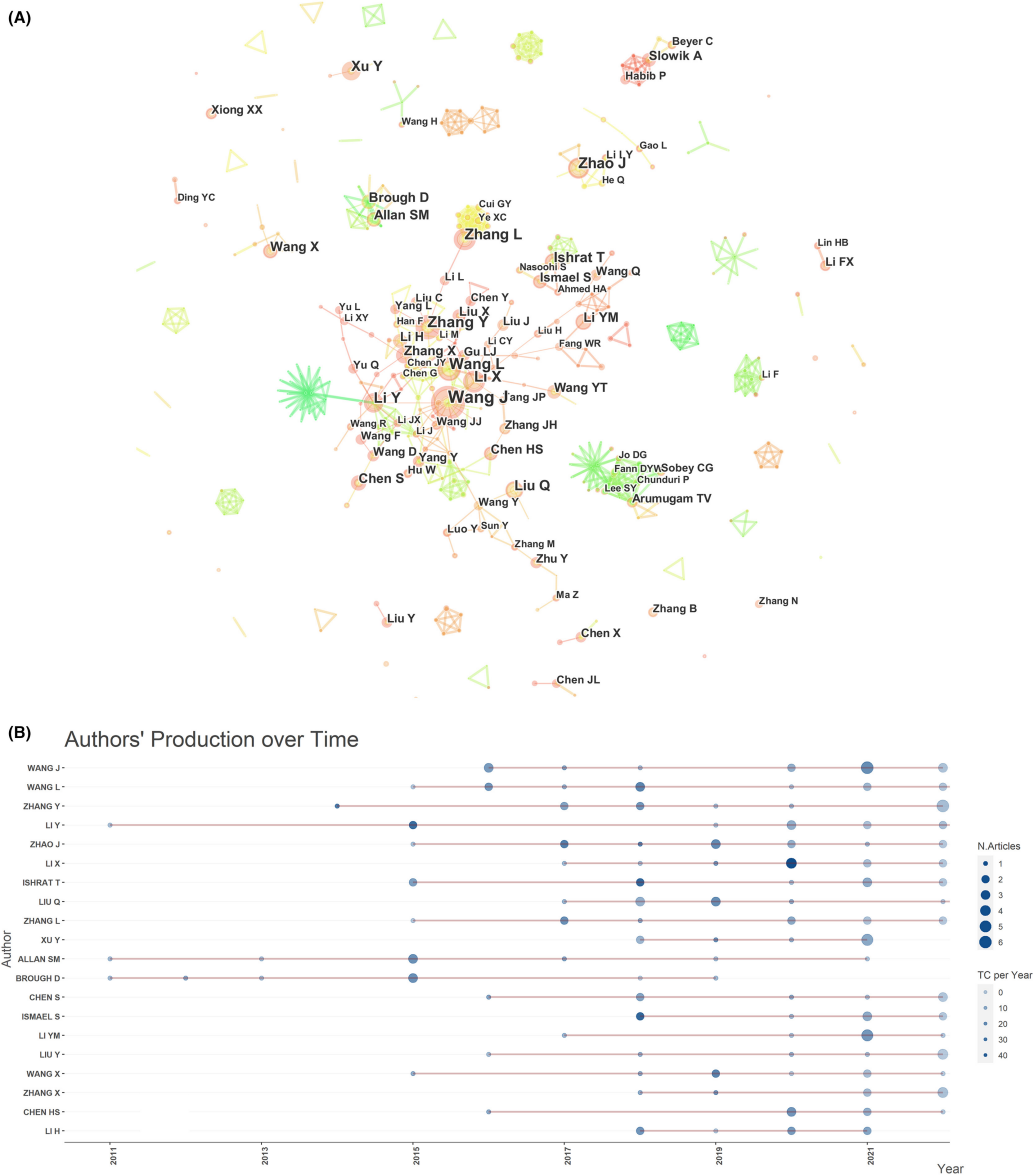
Network map of authors participating in NLRP3 research in ischemic stroke (A) and Top 20 authors' production over the time for NLRP3 research in ischemic stroke (B).

**FIGURE 7:**
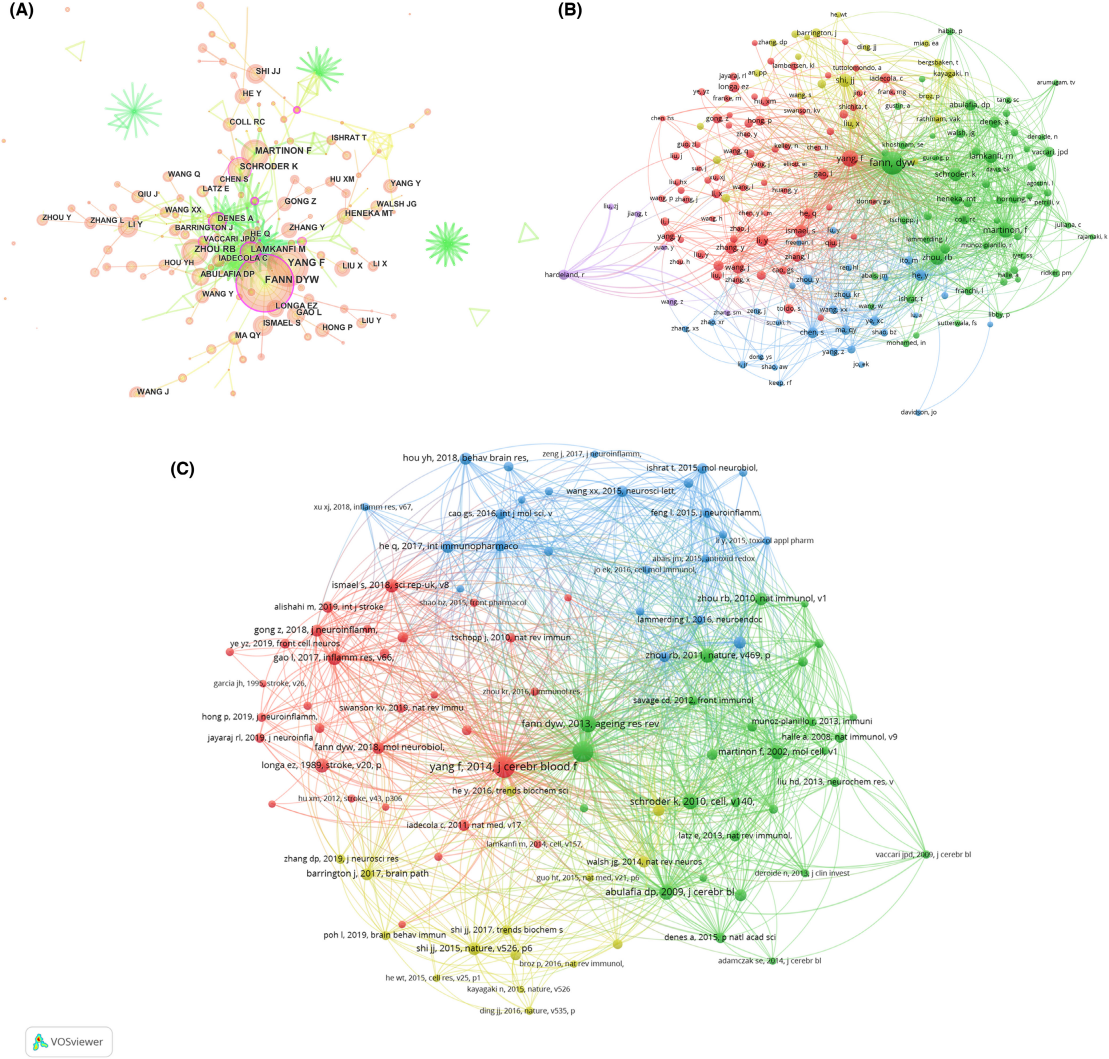
Network map of cocited authors (A, B) and cocited references (C).

A total of 3025 authors have published papers on NLRP3 research in ischemic stroke. Seven authors published over 11 articles. Wang J (*n* = 16, TGCS = 370) published the most papers, followed by Wang L (*n* = 12, TGCS = 408), Zhang Y (*n* = 13, TGCS = 552). Zhang L (*n* = 12, TGCS = 284), Li Y, and Zhao J (*n* = 12) (Table 4). The co‐authorship network is presented in Figure 6A. Figure 6B shows the results for the top 20 authors with the highest number of published papers. One of the most relevant authors was Wang J, who published 16 articles related to NLRP3 in the field of ischemic stroke. The red line in Figure 6B was the timeline of authors, and the size of the bubble was proportional to the number of publications in that year. The color intensity of the bubble was associated with the total number of citations of publications in that year. The darker the color, the higher the number of citations for that author's publications in that year. Figure 6B illustrated that more researchers began to study NLRP3 in the field of ischemic stroke in the last decade. The top 10 cited authors with the most citations were presented in Table 4. The top 5 cited authors were Arumugam (*n* = 6, TGCS = 949), Sobey (*n* = 5, TGCS = 859), Chunduri (*n* = 4, TGCS = 815), Fann (*n* = 4, TGCS = 815), Lee (*n* = 4, TGCS = 663). The remaining five top‐ranked authors have citations ranging from 542 to 632. Fann had the most co‐citation nodes and has active co‐citation relationships with Ismael, Longa, Gao, Hong, Liu, etc. (Figure 7A). Yang F had strong co‐cited relationships with Minutoli, Gao, Martinon, Wang, Zhang, etc (Figure 7B).

**TABLE 4:** Top 10 productive authors and most cited authors NLRP3 in researches in ischemic stroke.

Rank	Most productive authors				Most cited authors			
	Author	Records	TLCS	TGCS	Co‐cited author	Records	TLCS	TGCS
1	Wang J	16	42	370	Arumugam TV	6	190	949
2	Wang L	13	45	408	Sobey CG	5	161	859
3	Zhang Y	13	162	552	Chunduri P	4	159	815
4	Li Y	12	57	421	Fann DYW	4	159	815
5	Zhang L	12	73	284	Lee SY	4	120	663
6	Zhao J	12	109	534	Jo DG	4	79	632
7	Li X	11	36	356	Manzanero S	3	111	621
8	Ishrat T	10	78	419	Cheng YL	3	77	593
9	Liu Q	10	23	268	Zhang Y	13	162	552
10	Xu Y	9	15	214	Drummond GR	3	50	542

### 3.6 Publications and co‐cited reference analysis

We performed a statistical analysis of 601 publications and found that 34 documents had more than 100 citations. The top 10 most cited publications were shown in Table 5. The most cited publication was a review written by Walsh JG in *Nature Review Neuroscience* titled *Inflammasomes in the CNS* (TGCS = 444),^30^ followed by Xiong XY (TGCS = 353)^31^ and Fann (TGCS = 309).^32^ The network of co‐cited references was showed in Figure 7C, and the results illustrated that the document published by Yang F in *J cerebr blood f met* had the largest size of co‐citation nodes^33^ followed by Fann.^32^

**TABLE 5:** Top 10 most cited publications.

Title	First author	Year	Journal	TGCS	References
Inflammasomes in the CNS	Walsh JG	2014	Nat Rev Neurosci	444	^30^
Functions and mechanisms of microglia/macrophages in neuroinflammation and neurogenesis after stroke	Xiong XY	2016	Prog Neurobiol	353	^31^
Intravenous immunoglobulin suppresses NLRP1 and NLRP3 inflammasome‐mediated neuronal death in ischemic stroke	Fann DYW	2013	Cell Death Dis	309	^32^
NLRP3 deficiency ameliorates neurovascular damage in experimental ischemic stroke	Yang F	2014	J Cereb Blood Flow Metab	282	^33^
Bruton's tyrosine kinase is essential for NLRP3 inflammasome activation and contributes to ischaemic brain injury	Ito M	2015	Nat Commun	262	^34^
ROS‐Mediated NLRP3 Inflammasome Activation in Brain, Heart, Kidney, and Testis Ischemia/Reperfusion Injury	Minutoli L	2016	Oxid Med Cell Longev	238	^5^
Pathogenesis of acute stroke and the role of inflammasomes	Fann DYW	2013	Aging Res Rev	222	^35^
Evidence that NF–κB and MAPK Signaling Promotes NLRP Inflammasome Activation in Neurons Following Ischemic Stroke	Fann DYW	2018	Mol Neurobiol	194	^36^
AIM2 and NLRC4 inflammasomes contribute with ASC to acute brain injury independently of NLRP3	Denes A	2015	Proc Natl Acad Sci U S A	159	^37^
Curcumin attenuates glutamate neurotoxicity in the hippocampus by suppression of ER stress‐associated TXNIP/NLRP3 inflammasome activation in a manner dependent on AMPK	Li Y	2015	Toxicol Appl Pharmacol	156	^38^

### 3.7 Co‐occurrence of keywords and cluster analysis

**FIGURE 8:**
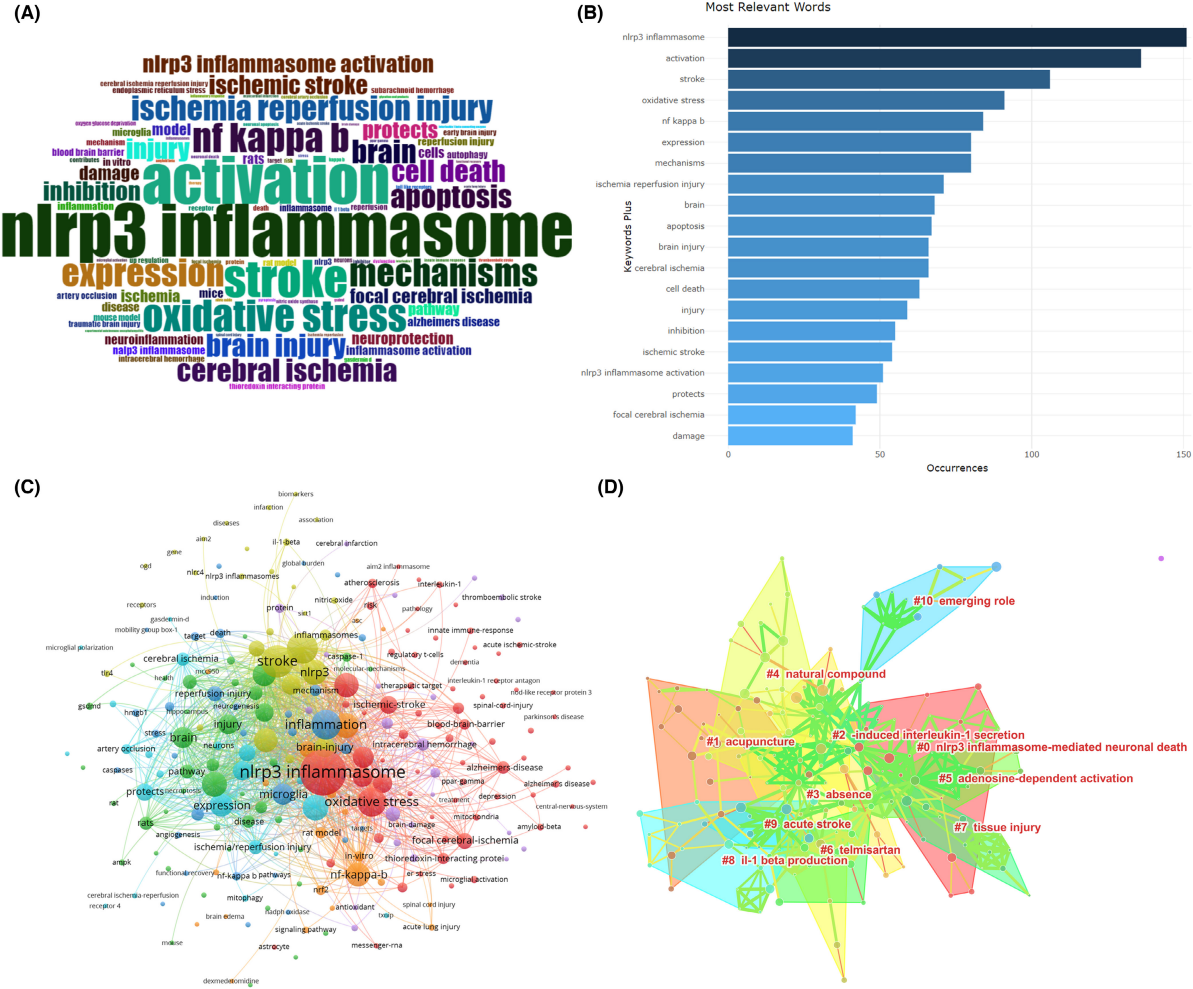
Word cloud of the most frequent keywords in NLRP3 research in ischemic stroke (A). Most relevant words in NLRP3 research in ischemic stroke (B). Co‐occurrence visualization of keywords (C). Clusters of keywords for NLRP3 research in ischemic stroke (D).

A total of 1584 keywords were identified, and 108 keywords appeared more than 10 times. Word cloud can visually illustrate the keywords and highlight the keywords with high frequency of occurrence. To quickly visualize the most prominent keywords in NLRP3‐related studies in the field of ischemic stroke, word cloud was generated for the extracted keywords. Figure 8A showed the word cloud of the keywords. The font size of a word represented its frequency of occurrence. The most frequent keyword was NLRP3 inflammasome, followed by activation, stroke, oxidative stress, and nf–κb (Figure 8B). Among the 1584 keywords analyzed in relation to studies on the topic of NLRP3 and ischemic stroke, a total of 222 keywords were identified as having appeared more than 5 times and the co‐occurrence between these 240 keywords was visualized (Figure 8C). The top 19 keywords with strongest citation bursts were shown in Figure S3. The results indicated that receptor antagonist, caspase 1, and il1 beta were the top three keywords with the strongest citation bursts. After naming the clusters with the terms extracted from the included publications, we found 11 clusters. The 11 clusters were named “#0 nlrp3 inflammasome‐mediated neuronal death”, “#1 acupuncture”, “#2 ‐induced interleukin‐1 secretion”, “#3 absence”, “#4 natural compound”, “#5 adenosine‐dependent activation”, “#6 telmisartan”, “#7 tissue injury”, “#8 il‐1 production”, “#9 acute stroke”, and “#10 emerging role” (Figure 8D). Additionally, the timeline view of clusters was showed in Figure S4.

## 4 DISCUSSION

This study is the first bibliometric study on NLRP3 in ischemic stroke research worldwide. The aim of this study was to perform a bibliometric analysis of research trends and hotspots in NLRP3 in ischemic stroke using the WoSCC database, HistCite Pro 2.1, VOSviewer, R package “bibliometrix”, and Citespace. We searched for 601 articles and reviews published from 2011 to 2022. The results show that there is an overall trend of more articles published during this 12‐year period. This suggests more researchers in the field of ischemic stroke disease research are becoming interested in the NLRP3 inflammasome.

Publications are scattered around the world. 1B depicts the geographic distribution of NLRP3 and ischemic stroke research publications. China had the most publications (*n* = 430), followed by the United States (*n* = 107), Germany (*n* = 26), and South Korea (*n* = 22). Among the top 10 countries/regions, China ranked first in terms of the number of published articles. And the first‐ranked TGCS and the top 10 institutions in terms of the number of publications are all from China, indicating that China has a great influence on this field in the past 12 years. Sun Yat Sen University (published 22 articles, cited 836 times) is the main representative. The article published in the journal of neuroinflammation in 2018 by Gong et al.^39^ from Sun Yat Sen University had the highest number of citations compared to the other articles in this unit, and they found that mitochondrial dysfunction caused the activation of NLRP3 inflammasome in the brain during ischemia–reperfusion injury, and the activation of NLRP3 was mainly concentrated in neuronal cells. Arumugam TV, from Australia, is a leader in NLRP3‐related research in the field of ischemic stroke and has a long history of research on immune‐inflammation in the field of neurological diseases such as ischemic stroke. His six publications in the field of NLRP3 and ischemic stroke have been cited 894 times, indicating the recognized outstanding contribution of Arumugam TV in this field. His most cited paper in this field is a 2013 article published in Cell death & disease that elucidates the inhibition of NLRP1 and NLRP3 inflammasome‐mediated neuronal death in ischemic stroke by intravenous immunoglobulin administration.^32^ His latest research in the field of ischemic stroke shows that delayed treatment with fluoxetine or atomoxetine plus limited voluntary running promotes motor recovery in mice after ischemic stroke.^40^

The two‐plot overlay of journals displays the distribution of NLRP3 in ischemic stroke research across journal fields and the citation relationships between different journal fields. In addition, articles associated with this field have been published in other fields, such as Neurology/Sports/ophthalmology, Medicine/Medical/Clinical, Physics/Materials/Chemistry, and Veterinary/Animal/Science. Notably, among the top 10 core journals, the *Journal of Neuroinflammation* and *International Immunopharmacology* currently have the highest number of publications and citations. According to Bradford's law, these two journals are the core journals in this field. These two journals publish the most cutting‐edge results and major breakthroughs in NLRP3 research in ischemic stroke. In recent years, the *Journal of Neuroinflammation* has focused on research related to inflammation in neurological diseases. For example, a recent article published in the *Journal of Neuroinflammation* found that intermittent theta‐burst stimulation improves motor function by inhibiting neuronal pyroptosis.^41^ An article published in *International Immunopharmacology* during the same period found that hirudin alleviated acute ischemic stroke through inhibition of NLRP3 inflammasome‐mediated neuroinflammation.^42^ Unlike the *Journal of Neuroinflammation*, which focuses on neuroinflammation research, *International Immunopharmacology* focuses on pharmacological breakthroughs. The top 2 most cited co‐cited journal are *Stroke* and *Journal of Cerebral Blood Flow & Metabolism*.

Among the top 10 most cited articles, the most cited is a review by Walsh JG published in 2014 in *Nat Rev Neurosci* about the role of inflammasomes in the nervous system.^30^ The second most cited review is a review by Xiong et al.^33^ published in 2016 that systematically describes the role of microglia/macrophages in neuroinflammation and neurogenesis after stroke, and that NLRP3 plays an important role in glial cell/macrophage polarization. Notably, NLRP3 regulation of glial cell/macrophage polarization in ischemic stroke has been extensively studied in the following years and has become a major hotspot in this field.^43^, ^44^, ^45^, ^46^ These extensive publications indicate that NLRP3 regulates microglia/macrophage polarization through the nf–κb pathway, specifically by promoting M1 macrophage polarization to exacerbate ischemia–reperfusion injury. Fann et al.^32^ published an article in *Cell Death & Dis* in 2013 elucidating NLRP1 and NLRP3 inflammasome meditation was involved in neuronal death during ischemic stroke, where inhibition of NLRP3 and NLRP1 alleviated neuronal death. In the following year, Yang et al.^33^ used NOX2 and NLRP3 knockout mice to elucidate that NOX2 knockout reduced NLRP3 expression and NLRP3 knockout mice showed better performance with less damage to the neurovascular unit. All these important findings laid an important foundation for subsequent studies related to NLRP3 in the field of ischemic stroke.

Reference co‐citations help to discover the knowledge base of the relevant research area. The co‐citation network graph of references was also examined. Among them, the most cited was the original article “NLRP3 deficiency ameliorates neurovascular damage in experimental ischemic stroke” published by Yang et al. in *J Cereb Blood Flow Metab* in 2014. The above article focused on the correlation between NLRP3 knockdown and vascular unit destruction caused by oxidative stress and other injuries in post‐ischemic brain tissue, reducing the level of the pro‐inflammatory factor IL‐1β by regulating knockdown of NLRP3, and thus reducing the inflammatory response and providing possible therapeutic approaches for new treatment options for stroke. This article is heavily cited, probably because the main background knowledge of NLRP3‐related studies in the field of ischemic stroke is related to the problem studied in this article

Cluster analysis allows the analysis of different clusters to determine the main research topics, frontier directions, and research progress in this research area. Hot spots are defined as scientific issues or topics discussed in a body of literature that are intrinsically linked to a particular time period. In bibliometrics, a co‐occurrence network graph of keywords can reflect hot topics. In this study, we performed a cluster analysis of keywords using CiteSpace software, and the results showed that the 1584 keywords were identified into 11 clusters. The 11 clusters were named “#0 nlrp3 inflammasome‐mediated neuronal death”, “#1 acupuncture”, “#2 ‐induced interleukin‐1 secretion”, “#3 absence”, “#4 natural compound”, “#5 adenosine‐dependent activation”, “#6 telmisartan”, “#7 tissue injury”, “#8 il‐1 production”, “#9 acute stroke”, and “#10 emerging role”. Cluster#0 mainly focuses on NLRP3 inflammasome‐mediated neuronal death, indicating the potential role of NLRP3 in neuronal damage. Cluster#1 focuses on acupuncture therapy on ischemic stroke. It has been reported that electroacupuncture reduces cerebral ischemic injury and neuroinflammatory response in stroke rats through α7nAChR‐mediated inhibition of the NLRP3 inflammasome.^47^ Additionally, acupuncture can suppress post‐stroke pain in animal models of ischemic stroke by upregulating the expression levels of SIRT1, inhibiting inflammasome activation, and downregulating the expression levels of IL‐18 (a downstream inflammatory factor of NLRP3).^48^ Acupuncture can also ameliorate cognitive impairment in stroke rats by modulating melatonin‐mediated autophagy and inhibiting NLRP3 inflammasome activation.^49^ Therefore, through our bibliometrics we found that in the last 2 years, acupuncture treatment has been carried out in the field of ischemic stroke and that acupuncture treatment may protect against ischemic stroke injury by decreasing NLRP3 expression. Cluster#2 and cluster#8 focus on the secretion of interleukin‐1. IL‐1 is known to be another downstream product of NLRP3 in addition to IL‐18.^50^ The specific role of IL‐1 as a downstream product of NLRP3 in ischemic stroke can be found in our review published recently.^51^ Cluster #5 focuses on adenosine‐dependent activation, suggesting that NLRP3 activation may be adenosine‐dependent. In fact, inactivation of endothelial adenosine A2A receptors attenuated ischemic brain injury and improved prognosis after stroke by inhibiting NLRP3 inflammasome activity.^52^ These findings suggest that cluster analysis provides a quick and straightforward way to gain a more comprehensive understanding of the direction of NLRP3 research in the field of ischemic stroke.

However, there are some limitations to our work. First, although WoSCC is considered the most important source of data for bibliometric analysis in science, we only included articles published in the WoSCC database related to NLRP3 in the field of ischemic stroke, which may not fully reflect the current status of all NLRP3 studies in ischemic stroke. Second, all included studies are in English, which may result in selection bias. Therefore, the findings may not be applicable to studies related to NLRP3 in ischemic stroke published in other languages.

## 5 CONCLUSION

With the help of HistCite, bibliometrix, CiteSpace, and VOSviewer; we have gained a better understanding of the research developments, hot spots, and future trends of NLRP3 in ischemic stroke over the past 12 years. The total number of publications in this field has increased rapidly. The leading countries are China and the USA, with numerous collaborations and communications between countries, institutions, and authors. The bibliometric analysis provides an objective and quantitative method to assess the trends and leading edge of NLRP3 in ischemic stroke, and provides important insights for researchers to understand the dynamics of the structure and timing of the field. This study also summarizes some specific mechanisms by which NLRP3 affects neuronal survival in ischemic stroke.

## AUTHOR CONTRIBUTIONS

Zhihong Jian, Xiaoxing Xiong, and Lijuan Gu designed this work. Hua Zhu and Yonggang Zhang wrote the manuscript. Hua Zhu, Shi Feng, Yina Li, and Yingze Ye downloaded and analyzed the data. All the authors contributed to the article revision and read and approved the submitted version.

## CONFLICT OF INTEREST STATEMENT

The authors declare no competing interests.

## Supporting information


Figure S1:

Figure S2:

Figure S3:

Figure S4:

Table S1:

Table S2:

Table S3:

## Data Availability

The original contributions presented in the study are included in the article/supplementary material, and further inquiries are available by contacting the corresponding/first authors.
