# Supplementary figures and images for: Trends in NLRP3 inflammasome research in ischemic stroke from 2011 to 2022: A bibliometric analysis
Source: CNS Neurosci Ther. 2023 Apr 23;29(10):2940–54. doi: 10.1111/cns.14232 (PMC10493663; doi:10.1111/cns.14232)

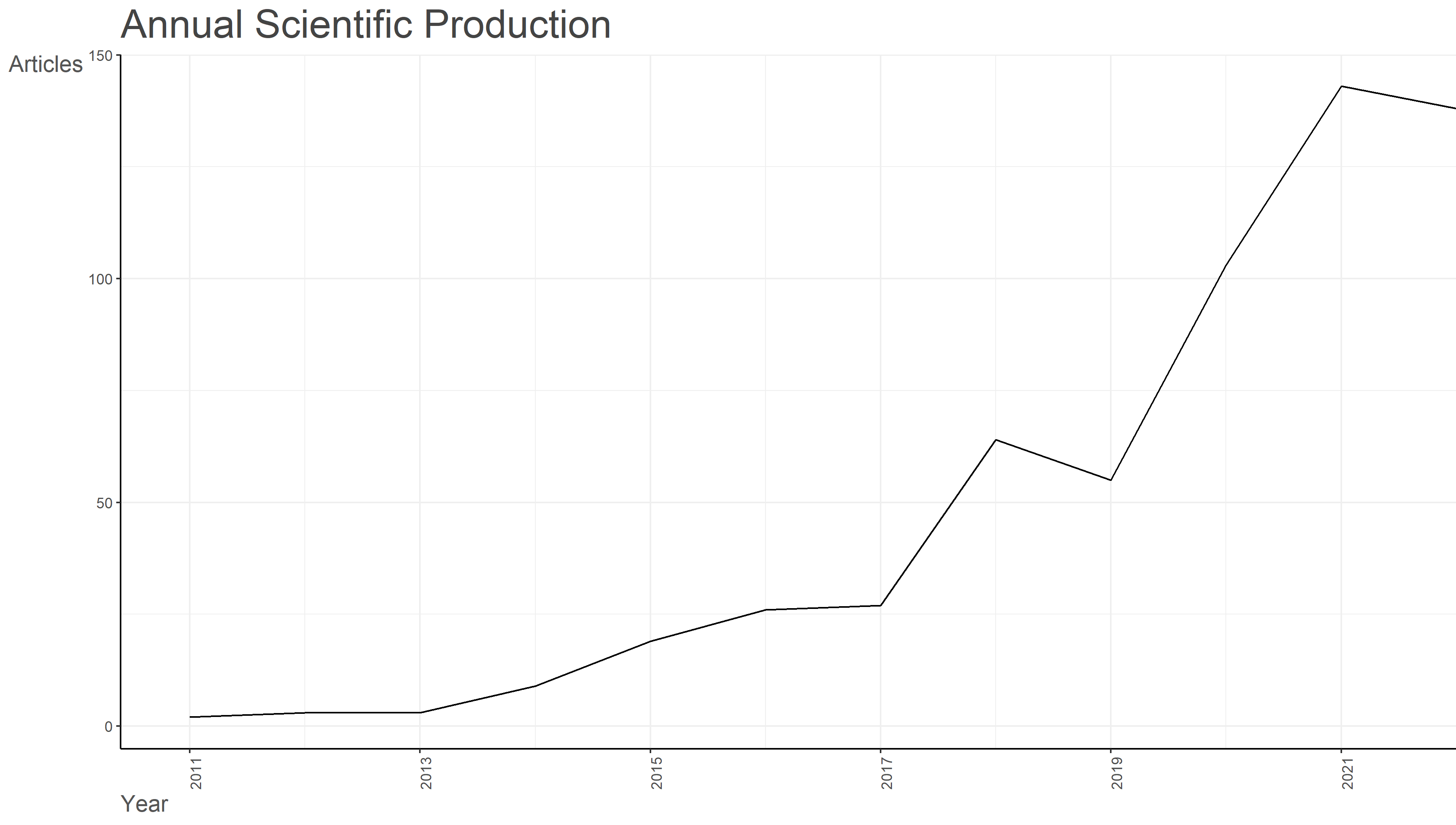

Supplement: Supplementary file 1 — Figure S1: [file CNS-29-2940-s006.png]

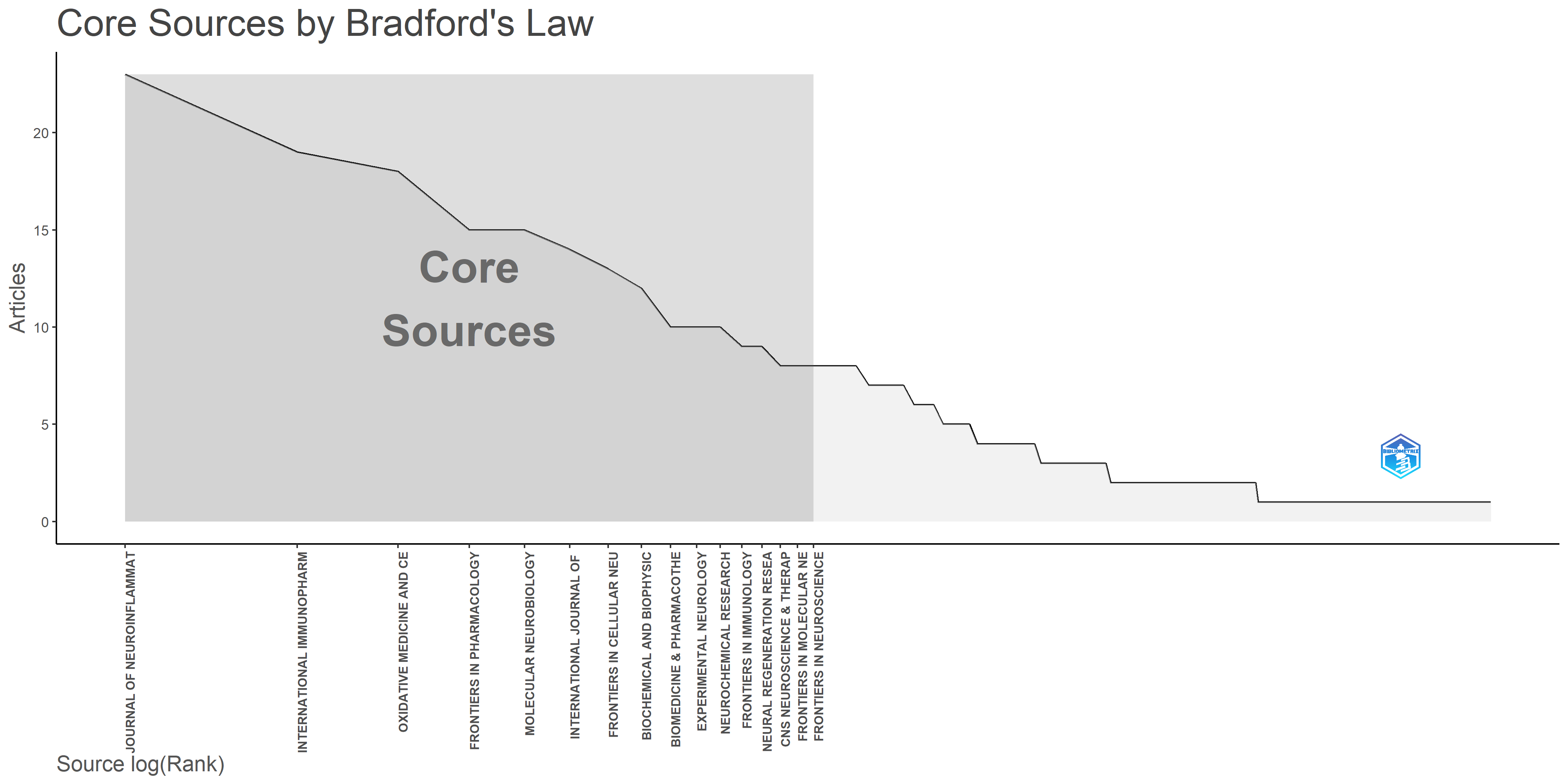

Supplement: Supplementary file 2 — Figure S2: [file CNS-29-2940-s007.png]

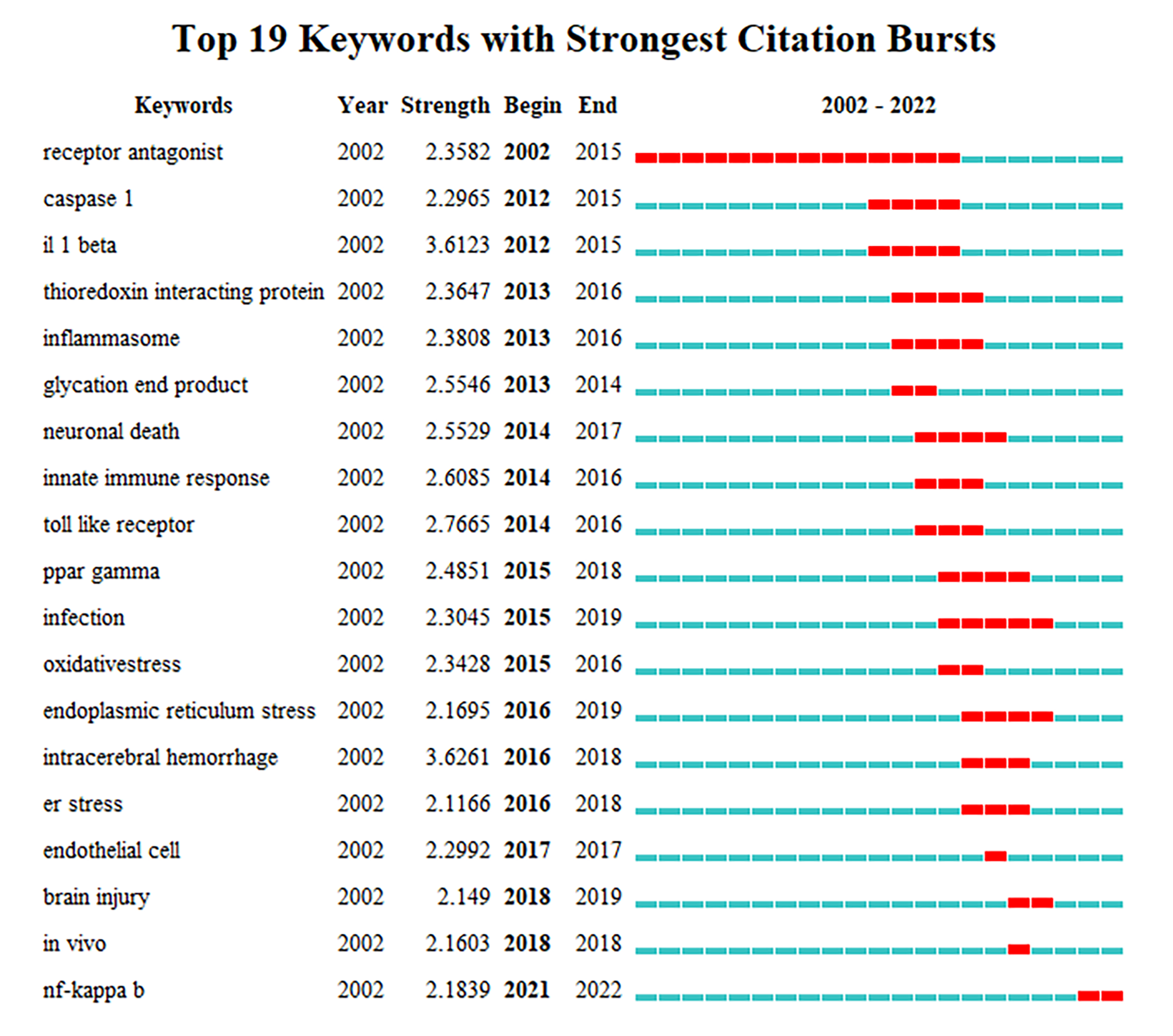

Supplement: Supplementary file 3 — Figure S3: [file CNS-29-2940-s002.png]

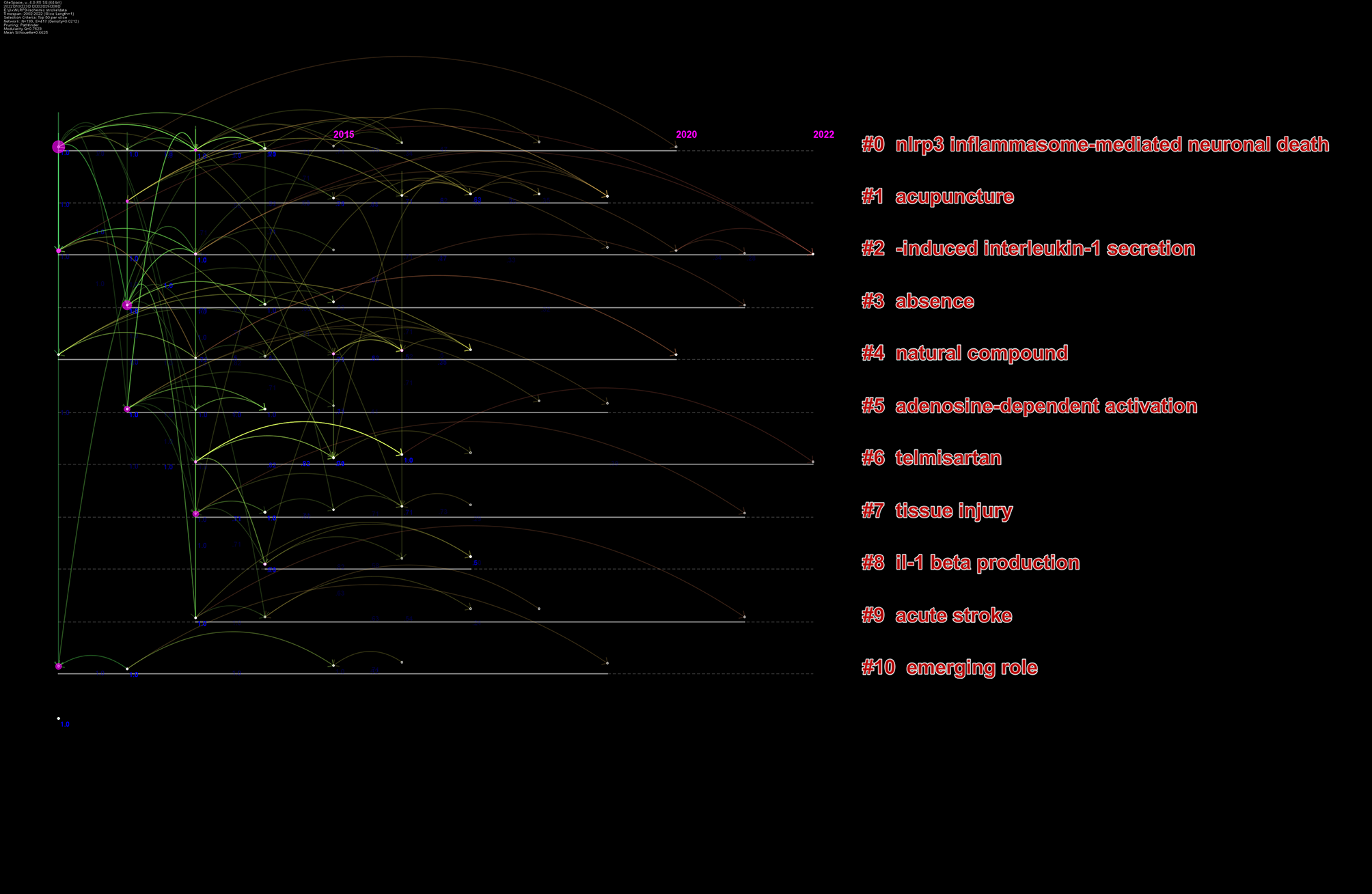

Supplement: Supplementary file 4 — Figure S4: [file CNS-29-2940-s001.png]
