# Supplementary material for: Trends in NLRP3 inflammasome research in ischemic stroke from 2011 to 2022: A bibliometric analysis
Source: CNS Neurosci Ther. 2023 Apr 23;29(10):2940–54. doi: 10.1111/cns.14232 (PMC10493663; doi:10.1111/cns.14232)
Supplement: Supplementary file 5 — Table S1: [file CNS-29-2940-s003.pdf]

Supplementary Table 1: Document type of NLRP3 researches in ischemic stroke.

Rank	Document Type	Records	TLCS	TGCS
1	Article	453	1343	11206
2	Review	141	386	5500
3	Article; Early Access	5	0	5
4	Article; Proceedings Paper	1	0	23
5	Review; Book Chapter	1	1	183
