# Supplementary material for: Trends in NLRP3 inflammasome research in ischemic stroke from 2011 to 2022: A bibliometric analysis
Source: CNS Neurosci Ther. 2023 Apr 23;29(10):2940–54. doi: 10.1111/cns.14232 (PMC10493663; doi:10.1111/cns.14232)
Supplement: Supplementary file 6 — Table S2: [file CNS-29-2940-s005.pdf]

Supplementary Table 2. Annual productions in NLRP3 related researches in ischemic stroke.

Rank	Publication Year	Recs	TLCS	TGCS
1	2011	2	5	94
2	2012	3	3	216
3	2013	3	84	576
4	2014	9	228	1525
5	2015	19	199	1296
6	2016	26	223	1952
7	2017	27	302	1623
8	2018	64	234	3212
9	2019	55	223	2248
10	2020	103	115	2357
11	2021	143	98	1515
12	2022	137	16	296
13	2023	5	0	2
14	Unknown	5	0	5
