# Supplementary material for: Trends in NLRP3 inflammasome research in ischemic stroke from 2011 to 2022: A bibliometric analysis
Source: CNS Neurosci Ther. 2023 Apr 23;29(10):2940–54. doi: 10.1111/cns.14232 (PMC10493663; doi:10.1111/cns.14232)
Supplement: Supplementary file 7 — Table S3: [file CNS-29-2940-s004.pdf]

Supplementary Table 3. Word collaboration NLRP3 related researches in ischemic stroke

From	To	Frequency
AUSTRALIA	INDIA	1
CANADA	FRANCE	2
CANADA	JAPAN	1
CANADA	PAKISTAN	1
CHINA	AUSTRALIA	5
CHINA	CANADA	6
CHINA	GERMANY	1
CHINA	INDIA	1
CHINA	IRAN	2
CHINA	IRELAND	1
CHINA	JAPAN	1
CHINA	KOREA	3
CHINA	PAKISTAN	1
CHINA	POLAND	1
CHINA	RUSSIA	1
CHINA	SAUDI ARABIA	3
CHINA	SINGAPORE	2
CHINA	SPAIN	1
CHINA	SWEDEN	2
CHINA	UNITED KINGDOM	2
CHINA	USA	43
EGYPT	IRAQ	1
EGYPT	NIGERIA	1
FRANCE	BELGIUM	1
GERMANY	AUSTRALIA	2
GERMANY	BELGIUM	1
GERMANY	CANADA	1
GERMANY	FRANCE	1
GERMANY	IRAN	1
GERMANY	ITALY	2
GERMANY	KOREA	2
GERMANY	MEXICO	1
GERMANY	NETHERLANDS	2
GERMANY	RUSSIA	1
GERMANY	SINGAPORE	3
GERMANY	SLOVAKIA	1
GERMANY	SPAIN	2
GERMANY	SWITZERLAND	1
GERMANY	UNITED KINGDOM	1
INDIA	SAUDI ARABIA	1
IRAN	AUSTRALIA	1
IRAN	CANADA	1
IRAN	POLAND	1
IRAN	UNITED KINGDOM	1
IRAQ	NIGERIA	1
ITALY	GREECE	1
ITALY	INDIA	1

ITALY	NETHERLANDS	1
ITALY	POLAND	1
ITALY	ROMANIA	1
ITALY	UNITED KINGDOM	1
KOREA	AUSTRALIA	5
KOREA	SINGAPORE	5
KOREA	SWEDEN	1
KOREA	UNITED KINGDOM	1
MEXICO	SLOVAKIA	1
ROMANIA	GREECE	1
ROMANIA	HUNGARY	2
SINGAPORE	AUSTRALIA	6
SINGAPORE	BELGIUM	1
SINGAPORE	FRANCE	1
SPAIN	HUNGARY	1
SPAIN	MEXICO	1
SPAIN	NETHERLANDS	1
SPAIN	RUSSIA	1
SPAIN	SLOVAKIA	1
UNITED KINGDOM	AUSTRALIA	1
UNITED KINGDOM	HUNGARY	1
UNITED KINGDOM	INDIA	1
UNITED KINGDOM	RUSSIA	1
UNITED KINGDOM	SPAIN	2
USA	AUSTRALIA	3
USA	BRAZIL	1
USA	CANADA	2
USA	GERMANY	1
USA	GREECE	1
USA	HUNGARY	2
USA	INDIA	4
USA	IRAN	5
USA	ITALY	3
USA	JAPAN	2
USA	KOREA	6
USA	MEXICO	1
USA	NORWAY	1
USA	POLAND	1
USA	ROMANIA	3
USA	RUSSIA	1
USA	SINGAPORE	4
USA	SPAIN	2
USA	SWEDEN	1
USA	UNITED KINGDOM	1
